# Malingering of Psychotic Symptoms in Psychiatric Settings: Theoretical Aspects and Clinical Considerations

**DOI:** 10.1155/2022/3884317

**Published:** 2022-04-21

**Authors:** Val Bellman, Anisha Chinthalapally, Ethan Johnston, Nina Russell, Jared Bruce, Shazia Saleem

**Affiliations:** ^1^University of Missouri Kansas City, Department of Psychiatry, Kansas City, MO, USA; ^2^University of Missouri Kansas City School of Medicine, Kansas City, MO, USA; ^3^University of Missouri Kansas City School of Medicine, Department of Biomedical and Health Informatics, Kansas City, MO, USA

## Abstract

Malingering is the intentional production of false or grossly exaggerated physical or psychological symptoms motivated by external incentives. Although the Diagnostic and Statistical Manual of Mental Disorders (DSM–5) does not list malingering in its diagnostic section and therefore does not identify it as a formal mental disorder, malingering and verified mental illness commonly coexist. Some subtypes of feigning behaviors, such as partial or pure malingering, dissimulation, and false imputation, can be suspected when patients have marked discrepancies between reported stressors and objective findings. The article discusses these three theoretical concepts with their possible clinical aspects, illustrating each phenomenon by clinical case with self-reported and/or observed psychotic symptoms. We summarized relevant findings and provided a review of clinical considerations that physicians can use to aid in the evaluation of psychotic symptoms in the context of those three concepts.

## 1. Introduction

Malingered psychosis involves the intentional falsification of psychiatric symptoms with a motive that generates tangible external benefits for the presenting patient [1]. Since the term “psychosis” encompasses a broad range of clinical presentations, malingering patients frequently choose to falsify psychotic symptoms rather than another type of disorder in order to gain external benefits [2] and create a diagnostic dilemma for clinicians who, by awarding benefit of the doubt, tend to group malingering with factitious disorders and similar clinical phenomena [3–5]. Dissimulation stands for a patient's attempts to conceal or minimize psychiatric or other clinical symptoms to pretend to be psychiatrically stable or otherwise healthy. Finally, false imputation occurs when the patient “ascribe real symptoms to a cause the individual knows is unrelated to the symptom” ([6], p. 14).

Although malingered psychosis has been well studied in the past, there are very few cases reported of patients presenting with psychotic symptoms in the context of dissimulation and false imputation. We attempted to describe these behaviors, discussed theoretical concepts, and summarized the literature to give a clear picture of the state of knowledge on the subject.

## 2. Methods

We discuss three cases of patients who attempted to misrepresent their symptoms to achieve their personal goals. This study was approved by the Institutional Review Board (IRB) of the University of Missouri Kansas City. The need for informed consent was waived by the ethics committee, due to the retrospective nature of the study and the nature of the disorder. All three charts of patients with various forms of malingering were identified and reviewed by the research team.

Extensive evaluation of available records was conducted in order to identify patients' external goals and underlying motives. Warning flags for malingering of psychotic symptoms included past episodes of similar behaviors, persistent noncompliance during prescribed evaluation or treatment, and striking inconsistency between objective findings, direct/indirect observations, and stated symptoms. The patient's psychosocial profiles, underlying motivations, and variable experiences with psychiatric services predetermined their clinical and socio-occupational outcomes.

## 3. Results

### 3.1. Case 1

Mr. H was a 19-year-old, single, unemployed Caucasian male who was transferred to the emergency department (ED) after attempting suicide by hanging himself with an article of clothing while incarcerated. The patient reported a history of continuous auditory hallucinations lasting more than two hours a day and described at least two unrecognizable voices telling him to kill himself and commit harmful or illegal acts “on command.” The nature of his auditory hallucinations was unclear, but they were described as being intense and threatening.

The patient continued to report experiencing persistent psychotic symptoms along with sleeplessness, despite contradictory nursing reports. In a follow-up evaluation, he reported that he was hearing voices saying “he is here and he wanted me to die,” but did not expand further upon this description. Due to the patient's changes in symptoms (e.g., dramatic worsening of psychotic symptoms in front of a new inpatient psychiatry team, well-organized behavior during leisure activities, and clear ability to make rational decisions), questionable perceptual disturbances, and lack of clinical improvement on medications, there was a high suspicion of malingering. Further analysis revealed that the patient had falsified information about his charges and purposefully influenced others in an attempt to be admitted to inpatient psychiatry and avoid incarceration.

### 3.2. Case 2

Mr. S is a 34-year-old, single, unemployed male with an unknown past psychiatric history, who was brought by the police for psychiatric evaluation and later admitted for inpatient psychiatric care. Prosecutors said he tried to kill his wife and friend because he thought she was cheating on him. On assessment, the patient was exceptionally polite, calm, and cooperative, denied having any mental health issues and tried to convince the examiner to accept his story. Although he attempted to present himself as mentally healthy (e.g., provided somewhat logical examples and explanations that could not be verified and appeared to be receptive to the feedback), his psychiatric status was impaired by somewhat tangential comments and superficial paranoid ideations. He reported that his “soul” instructed him to act in specific ways and appeared to be responding to internal stimuli on indirect observation. A review of collateral information revealed that the patient had been diagnosed with schizoaffective disorder and had multiple psychiatric admissions prior to that marriage. According to nursing reports, he repeatedly stated that “being perceived as crazy” and “being placed in a mental institution” were the worst things he could ever imagine. He repeatedly denied all psychiatric symptoms, demonstrated prosocial behaviors, and presented a dissimulating attitude, denying his past psychiatric issues. Occasionally, the patient responded to internal stimuli by looking around the room when no one was present or interacting as if someone or something else was present. Although he demonstrated the objective signs of an underlying psychotic process, his behavior was almost ideal and uneventful. After his release, the patient struggled with mental health before he was eventually arrested and incarcerated for drug offenses.

### 3.3. Case 3

Ms. F is a 37-year-old, single, unemployed, Caucasian female with a self-reported history of anxiety and vague auditory and visual hallucinations (AVH) who presented to the community clinic for a psychiatric evaluation. She stated she was not treated by a mental health professional but, instead, was given a recurring prescription for oral quetiapine 100 mg PO by her primary care provider. She also stated she would like to apply for Social Security Disability and needs to provide medical documentation to “prove my disability case.”

A review of the patient's symptoms was significant for intermittent auditory hallucinations suggesting harm, threats to safety, vague persecutory delusions about people being “out to get her.” Overall, her mental status examination was relatively unremarkable. She denied any previous psychiatric admissions, medical problems, or overuse of any recreational substances. In fact, she did not demonstrate any notable signs of psychosis and displayed a high level of insight into her illness. She refused to provide a urine sample that the nurse could send to a lab for testing, informed the provider that she had looked online for schizophrenia-related information, and appeared to be convinced about a self-diagnosis. She also repeatedly requested that her paperwork be sent to the local SSA office for “disability verification.”

Collateral information revealed a four-year history of paranoia and persecutory delusions exclusively in the context of daily methamphetamine use. She was selling her quetiapine on the street and tried to collect disability benefits after seeing several providers. Her Social Security Disability Insurance (SSDI)/Supplemental Security Income (SSI) application was eventually dismissed, as they could not determine the validity of her claims.

## 4. Discussion

Resnick [7] suggested that malingering may involve deliberate exaggeration of existing psychopathology (partial malingering); production of fake symptoms (pure malingering); deliberate misattribution of genuine symptoms to another cause (false imputation); or a combination of the three. No empirical studies have systematically examined Resnick's malingering subtypes. The true prevalence of these subtypes in the clinical setting is also unknown. Kleinman and Stewart [8] stated that partial malingering is more common, while pure malingering is quite rare [9]. Furthermore, false imputation has not been formally studied as frequently as other forms of malingering.

While this classification may be considered outdated, feigning behaviors can be clinically variable and may include difficult clinical presentations that do not clearly represent malingering or any specific subcategory. For example, Rogers [10] indicated that the term dissimulation should also be considered when describing an individual who is deliberately distorting or misrepresenting psychological symptoms.

While some experts describe dissimulation as deliberately distorting nonexistent problems or exaggerating real symptoms [11], most authors believe that patients may actually dissimulate or downplay psychopathological difficulties to achieve secondary gain [12, 13]. Some individuals can underreport their symptoms even in the correctional system to appear psychologically healthy, despite their possible self-interest [12]. In false imputation, individuals may associate their symptoms with causes that are completely unrelated to achieving their personal goals. Intentional deception is an element of these theoretical concepts; however, motivation is not always clear.

Research in the field of forensic psychiatry illustrates that malingering is situation-dependent and dynamically constructed rather than static and rigid response strategy [14, 15]. Although the motivation for malingering (including false imputation) is typically objective (e.g., being admitted to a psychiatric hospital to obtain prescription medications, free shelter, food, financial gain due to disability, and protection from the legal system), factors including family history of mental illness, childhood trauma, past behavioral disturbances, and treatment history should also be considered [16, 17]. Rogers [10] and Resnick [7] also emphasized that the existence of both external goals and internal motives is neither contradictory nor mutually exclusive. Patients with “iatrogenic malingering” typically present with “mislabeled, embellished, or feigned” claims of suicidality and auditory hallucinations in order to gain access to more comprehensive care and local psychosocial services ([18] p.253).

In dissimulation, the main goal for denying psychiatric symptoms could be obtaining employment, gaining discharge from a mental institution, being released from a correctional facility, or being awarded custody of a child [19–23].

McDermott et al. [24] found that differing incentives influence the likelihood of malingered psychotic symptoms. They found that 17.5% of defendants feigned psychotic symptoms so as to be declared incompetent to stand trial, while 64.5% malingered to receive psychiatric service instead of serving prison terms. The highest rates of imitating psychotic symptoms were found in other general categories, such as obtaining more desirable housing or obtaining prescription medications [24].

Commonly discussed motives and types of secondary gain associated with malingering are summarized in Table 1.

The frequency and intensity of malingering in psychiatric settings can vary considerably. Jelicic et al. [31] and others have reported that patients with previous experience in inpatient psychiatric settings can credibly feign psychotic disorders [3, 31]. However, this contradicts earlier results by Cornell and Hawk [32], who did not think it was possible to outwit professionally trained evaluators. Given substantial substance abuse history and various pathological personality traits, patients may exhibit fluctuating psychopathological symptoms. Although exacerbation of psychosis is not an uncommon clinical syndrome, it is important for physicians to consider all causes in patients experiencing psychotic symptoms [33].

## 5. Malingered Psychosis

Psychosis encompasses a broad range of clinical presentations and can be used purposefully by malingerers to gain external benefits and create a diagnostic dilemma for clinicians. Standardized criteria typically portray malingerers as having problematic personality traits. However, these individuals may also be trying to adapt to challenging circumstances with limited resources. [34]. There is a considerable heterogeneity in symptom profiles among adults with self-reported and/or suspected psychotic symptoms. Moreover, some individuals with schizophrenia may keep feigning or exaggerating the severity of their symptoms for the same external (secondary) gains [35]. Thus, the provider should utilize various strategies for determining the presence of malingering.

### 5.1. Auditory Hallucinations

While auditory hallucinations (AH) are considered “classic” psychotic symptoms, they are also associated with bereavement, trauma, and intense emotions, borderline personality disorder, insomnia, traumatic brain injury (TBI), and organic brain lesions [36–38]. De Marchi and Balboni [39] outlined some characteristics of AH that are consistent with established psychotic conditions. For example, a single sensory mode is common in the manifestation of a psychotic disorder, while multiple and/or simultaneous modes may be considered signs of malingering [40]. Pollock [41] also suggested that malingered hallucinations tend to be exaggerated and dramatic. Interestingly, patients with self-reported monosymptomatic auditory verbal hallucinations should be assessed for “iatrogenic malingering” [18].

Kumar et al. [4] added that true AH are typically clear and conversational, while vague and threatening ones are more likely to be feigned. Malingerers often describe their AH using swear words or hurtful or socially unacceptable language, characterized by situationally inappropriate obscenities [6]. For example, the patient described in case #1 reported hearing voices whose thematic content included repeated insults, threats, and terms of abuse.

The possibility that phenomenologically rich auditory verbal hallucinations can be voluntarily controlled by patients with primary psychotic disorders is also discussed by several authors [42, 43], while patients with malingered AH cannot fully expand on what they do to dampen voices. Specifically, Mr. H (case #1) did not seem to be emotionally disturbed by self-reported “unbearable” voices (e.g., did not try to reduce the intensity of the voices through seeking company or taking PRN medications) as it would be expected from patients with truly destressing auditory hallucinations [44].

There is substantial heterogeneity in the clinical presentation of psychotic disorders, both within and across diagnostic categories. Individuals with psychosis can present quite differently in terms of the description of experienced auditory hallucinations [45], associated personality traits, and/or the domains of functional impairment. Despite this, many professionals still assume that “true” auditory hallucinations are experienced as clear, negative and/or derogatory, loud voices, intermittent rather than constant, and coming from outside the head. McCarthy-Jones and Resnick [45] emphasized that this description of AH is outdated and no longer valid, though it is often still used to refer to AH as a “first-rank” symptom of schizophrenia. From the diagnostic and clinical perspectives, an important unresolved question is the degree to which heterogeneity in auditory hallucinations reflects fundamental differences or similarities in the etiology and potential outcomes. Failing to consider such heterogeneity may lead to inappropriate assumptions among mental health providers and may potentially influence treatment efficacy.

Cultural differences are a significant factor to consider when working with diverse patients in mental health. Luhrmann et al. [46] indicated that AH reported by participants from the United States tend to be harsher and lead to more psychotic diagnoses. While data on that topic are still limited, cultural aspects should always be considered [46].

### 5.2. Visual Hallucinations

Visual hallucinations (VH) are the most common symptom reported by malingerers [10]. They may include formed images (e.g., people) or unformed images (e.g., flashes of light or shadows) in full color, while malingered VH may more commonly be described as “black and white” or single-color perceptual disturbances [47]. VH in those with primary psychosis tend to involve visions regarding family members, religion, and/or animals [48]. Psychotic VH can be brief or prolonged, usually described as colorful and involving normal-size objects, and usually do not disappear if the eyes are closed/open or if the lights of the room are turned off/on [49]. Gauntlett-Gilbert and Kuipers [50] also indicated that VH include humanoids, sometimes other objects, and usually lead to overwhelming response and persistent fear. Given that many patients present with a history of substance use, it is important to note that drug-induced psychosis is often associated with VH and paranoid ideation [51]. Drug-induced VH are also usually seen better when eyes are closed [47]. Patients are usually aware of symptoms and demonstrate fair insight into the drug-induced nature of psychosis. Further, perceptual disturbances appear only during periods of substance use, withdrawal, and/or sudden increase in substance potency [52]. Visual disturbances with atypical features are common in malingering.  Table 2 provides differential diagnosis and descriptions of visual hallucinations in different conditions that can mimic a primary psychotic process.

### 5.3. Delusions

A systematic review by Harris [35] reported that although malingerers may claim sudden onset, delusions tend to develop gradually over weeks or months. The duration of untreated delusions is directly associated with the time to respond to treatment [68]. Usually, as a patient's psychosis stabilizes, their delusional thoughts diminish in frequency and intensity. Although our patients had been treated with a second-generation antipsychotic (SGA) prior to inpatient admission, they still demonstrated vague delusional thought content and/or experienced persecutory delusions as prior to initiation of therapy. There is a direct correlation between the intensity of delusions and the level of disorganization among patients with schizophrenia, but the information regarding other psychotic disorders is still limited [69]. The content of delusional beliefs has a direct relationship to the patient's environment [70, 71] and their behavior is directly related to the specific delusions' content [72], while vague description and inconsistent behavior may be considered a possible sign of malingering [73, 74]. Harris [35] and Powers et al. [75] agreed that most psychotic patients have reasonably detailed accounts of their delusions and hallucinations. For example, Mr. S described an extremely rigid delusional system characterized by recurrent accusations of infidelity, searches for evidence, repeated interrogation of the partner, and sometime stalking.

Moreover, strongly held delusional beliefs can be distinguished from malingering by the confrontational reactions that patients may have toward the examiner questioning the content of their delusion [76]. This view has been supported by our observations when the patients appeared to be “normal” during the period of self-claimed psychosis and showed defensive hypersensitivity to questions during follow-up sessions.

### 5.4. Dissimulation

Dissimulation refers to a type of deception in which an individual tends to obscure the truth but does not present false information, and this has been a special focus of clinical attention [77]. Although dissimulation is sometimes listed as a malingering subtype, patients with malingered psychiatric symptoms often have recognizable and understandable goals, especially under the circumstances involved. However, dissimulation sometimes involves unclear and unrecognizable goals that can only be understood upon careful assessment. Dissimulation of psychotic symptoms can be very difficult to identify: the hallmark symptoms of “true” psychosis can vary and appear over a period of time/subside with time.  Table 3 sums up some relevant characteristics of malingering and dissimulation.

Dissimulation can be both positive (“faking good”) and negative (“faking bad”). People frequently demonstrate positive dissimulation in daily situations to increase their chances of getting a job or achieving their personal goals [78]. From the legal perspective, effecting “faking good” can affect various decisions regarding the legal commitment, proceedings, and release from police custody. In medicine, negative dissimulation is predominant and heterogeneous, as it includes feigning and exaggerating a wide range of physical and psychiatric conditions [79]. In psychiatry, dissimulation occurs mostly when patients tend to distort, minimize, and underreport their psychological, cognitive, or physical symptoms to present themselves as mentally stable individuals [77]. Most, if not all, dissimulation cases are driven by the motive of a secondary gain or rather a potential benefit in concealing information [80]. It is therefore important to look at the reasons for minimizing these symptoms and adopt instruments to detect dissimulations in clinical settings. Researchers have attempted to systematize multiple explanatory models and link them to specific conditions to explain the motivation behind dissimulations, but it should be clearly outlined that dissimulation in psychiatry is always motivated by internal or external gains rather than unintentional and/or unconscious simulation of symptoms. Patients may also withhold critical information about their psychopathology due to paranoid fears, external factors, or desires to appear normal [81].  Table 4 summarizes the most common conditions associated with dissimulation in the field of psychiatry.

### 5.5. What about False Imputation?

False imputation involves individuals who attribute genuine symptoms to a different cause than the original one. In other words, people ascribe symptoms to unrelated causes to gain different advantages. It may, however, involve unconscious processes and may not always be a part of malingering [82].

For instance, a person may claim injuries from a car accident during personal injury litigation, although the pain originates from an unrelated fall. Another example would be a woman who demonstrates signs and symptoms of psychosis because of substance use could be deliberately and falsely attributed to schizophrenia as described in case #3. According to Resnick's model of malingering, individuals' ability to falsify the symptoms may be directly related to their past experiences with the feigned condition [7]. In psychiatric settings, pure malingerers will have to engage in more extensive exacerbation or feigning of psychotic symptoms, since they do not experience any genuine symptoms of primary thought disorder. Interestingly, “false imputators” who have already experienced the symptoms of psychosis would not need to falsify the symptoms as dramatically as pure malingerers. Given the complexity of many psychiatric disorders, false imputation is posited to be the most difficult malingering subtype to detect. It is exceptionally difficult to detect the false imputation of psychotic symptoms, especially when collateral information is not available [83]. Although data on the false imputation of psychotic symptoms are quite limited, it may resemble the same false imputation of PTSD-like symptoms.

In theory, individuals using false imputation genuinely suffer from underlying psychotic disorders, but simply ascribe their symptoms to different conditions (e.g., emotional brake, stress, hormones, and genetics) for the purposes of a legal claim. Similarly, patients with a previous diagnosis of PTSD also tend to attribute their symptoms to a different trauma [83]. There are some overlapping symptoms of both PTSD and psychosis that make diagnosis challenging, but the validity of patients' claims regarding psychotic symptoms can sometimes be verified by observing patients' behaviors (e.g., responding to internal stimuli in the case of a primary thought disorder).

A formal diagnosis of the patient in the past cannot be considered an effective indicator of false imputation—many patients with a history of primary psychotic disorder may relapse or develop other symptoms associated with this condition. Likewise, in PTSD, the key to detecting the false imputation of psychotic symptoms is the timeline of the symptoms [7, 84]. Medical records and collateral information should always be considered in the detection of false imputation.

## 6. Limitations

These case reports can be prone to bias, limiting its generalizability to larger populations of patients. This research relies on accuracy of written records and recall of individuals while the impact of confounding factors may not be fully assessed. Lack of agreement between objective and subjective measures in the evaluation of malingered psychosis can negatively affect the validity of the research. Moreover, it can be seen as focusing on misleading aspects of patients' presentations. Given the nature of these conditions, we recognize that patients' symptoms may have other explanations [85].

## 7. Conclusion

We believe this article sheds light on a previously understudied area of forensic and clinical psychiatry. All subtypes of malingering are related to specific objectives in particular contexts. When malingering is suspected, clinicians should not dismiss all the symptoms as “faked.” The data presented in this paper show that feigning of symptoms is not necessarily malingering. Any attempt to distort or misrepresent facts such as defensiveness may be considered a form of deception, but malingering should not be diagnosed if deception, dissimulation, or false imputation are present, as those terms are associated with other psychiatric disorders. These cases clearly demonstrate that behavioral patterns such as dissimulation and/or false imputation have not received adequate attention in our professional training and clinical practice, thereby leaving many providers unprepared when they encounter them.

At this point, no one evaluation methodology can be used in isolation to diagnose malingering [86]. Given the complexity of the malingering assessment process, it is critical for an examiner to explore all possible explanations for patient behaviors to avoid incorrectly classifying and potentially stigmatizing a patient as a malingerer [87]. The conceptualization of malingering in diagnostic tools such as the DSM-5 can be criticized for having poor reliability and validity [88, 89].

The review of published data suggests that the behavioral assessment of malingered psychosis is well described in the literature. We also need additional research to better understand how to best identify and treat patients with dissimulation and false imputation.

## Figures and Tables

**Table 1:** Types of secondary gain in the malingering of psychotic symptoms.

Motivation	Frequencies of malingering	References
Evade criminal prosecution or protection from the legal system	30% of disability evaluations, 29% of personal injury evaluations, 19% of criminal evaluations, and 8% of medical cases	Mittenberg et al. [25]
Avoid work, military service, or personal obligations	10–12% of psychiatric inpatients	Chandran et al. [3] Harris & Resnick [26]
Obtain controlled substances and/or psychotropic medications and/or intentional admission to psychiatric facility	13% of patients in the ER	Yates et al. [27]
Obtain food and/or housing	No data available	Brady et al. [28]
Financial compensation	No data available	Waite & Geddes [29]
Attention-seeking motive	No data available	Oke et al. [30]

**Table 2:** Differential diagnosis of visual hallucinations.

Visual hallucination characteristics	Possible cause	References
VH are usually simple, with appropriate insight Valsalva-like maneuvers can trigger VH	Retinal pathology and/or retinal traction	[53]
Release hallucinations, simple, and complex in nature, with intact insight and a history of visual acuity loss	Charles Bonnet syndrome	[54]
Simple VH/disturbances such as flickering, uncolored, unilateral zigzag linear changes in the center of the visual field that gradually progress toward the periphery, often leaving a scotoma	Migraine with aura	[55]
Simple, brief, and consistent for each patient; usually consist of small, brightly colored spots or shapes that flash	Epilepsy or seizure disorder	[56, 57]
Seeing objects move when they are actually still and seeing complex scenarios of people and items that are not present	Dementia with Lewy bodies	[58]
Simple and complex VH with acute disturbance of consciousness and diminished ability to sustain attention	Delirium	[59, 60]
VH of crawling insects	Cocaine and methamphetamine intoxication/withdrawal	[61–63]
Shadows, flashing lights, and moving objects	Cocaine intoxication/withdrawal	[62, 63]
VH with some type of animal life such as “animals on the walls”	Alcohol-induced hallucinations	[64]
VH of colored patterns, geometric shapes, and figures of animals and people; size distortion and the feeling of fantasy; hypnagogic hallucinations	Hallucinogens	[65]
VH including trailing of moving images, geometric hallucinations, flashes of color, and halos around objects	Hallucinogen-persisting perception disorder	[66, 67]

**Table 3:** Clinical aspects of simulation and dissimulation.

Characteristics	Simulation (malingering)	Dissimulation
Symptom severity	Severe	Minimal
Self-reporting of the symptoms	Consistent with potential overendorsement of certain symptoms	Consistent with potential minimization/underreporting of symptoms
Contradictory or unusual symptoms	Likely	Unlikely
Obvious vs. subtle presentation	More obvious symptoms	Vague, subtle symptoms
Manifestation and/or progression of symptoms	Sudden onset of symptoms	Sudden resolution
Self-harming statements or actions	Unlikely	Unlikely
Self-report vs. observation inconsistency	Possible	Likely
Endorsement of highly specific symptoms	Likely	Unlikely

**Table 4:** Dissimulation in connection to specific conditions and underlying motivations [10].

Disorder	Explanatory model	Characteristics associated with dissimulation and underlying motivations
Conduct disorder	Criminological	Poor impulse control and unpredictability
Reactive attachment disorder	Pathological Adaptational	Secondary to extreme abuse and abandonment Compensatory mechanisms in social situations
Factitious disorders	Criminological Pathological Adaptational	Secondary to antisocial behavior/psychopathy Rigidity Financial motivations
Substance abuse	Criminological Pathological Adaptational	Secondary to antisocial behavior/psychopathy Self-medication/comorbidity Avoiding adult responsibilities/escapism
Eating disorders	Pathological	Maintaining control/rigidity, distorted body image
Paraphilias	Criminological Pathological	Luring victims/maintaining offending Own abuse history leads to poor boundaries
Psychopathy	Criminological	Instrumental/game-playing/poor impulse control
False memory syndrome	Criminological Pathological Adaptational	Secondary to antisocial behavior/psychopathy Regression/repression/avoiding responsibilities Financial motivations (evaluating malingering)
Child custody	Criminological Pathological Adaptational	Extortion/lying to turn child against parent Rigidity/pathological denial or acknowledgment Denial of problems to remain with child
Chronic fatigue syndrome	Criminological Pathological Adaptational	Secondary to antisocial behavior/psychopathy Secondary to mental illness/comorbidity Financial motivations/receiving disability

## Data Availability

Data are available on request due to privacy/ethical restrictions.
